# Impairment of the Functional Status and Decrease in Albumin in Frail Older People After a COVID-19 Outbreak: A Descriptive Study in a Long-Term Care Facility in Chile

**DOI:** 10.3390/geriatrics10010001

**Published:** 2024-12-25

**Authors:** Lidia Castillo-Mariqueo, Alejandro Aedo Lagos, Lydia Giménez-Llort, Neftalí Guzmán Oyarzo

**Affiliations:** 1 Carrera de Kinesiología, Departamento de Procesos Terapéuticos, Facultad de Ciencias de la Salud, Universidad Católica de Temuco, Temuco 4780000, Chile; lcastillo@uct.cl; 2 Establecimiento de Larga Estadía para el Adulto Mayor (ELEAM) Santa Isabel de Traiguén, Traiguén 4730000, Chile; aedolagos.a@gmail.com; 3 Institut de Neurociències, Universitat Autònoma de Barcelona, 08193 Barcelona, Spain; lidia.gimenez@uab.cat; 4 Department of Psychiatry and Forensic Medicine, School of Medicine, Universitat Autònoma de Barcelona, 08193 Barcelona, Spain; 5 Laboratorio de Investigación en Salud de Precisión, Departamento de Procesos Diagnósticos y Evaluación, Facultad de Ciencias de la Salud, Universidad Católica de Temuco, Temuco 4780000, Chile

**Keywords:** COVID-19, disability, frailty, functionality, SARS-CoV-2, physiological function, LTC

## Abstract

**Introduction.** Frailty is a common condition among older individuals and is associated with increased vulnerability to adverse health outcomes. The COVID-19 pandemic further highlighted the impact of viral infections on frail populations. The present work aimed to determine frailty, functional and cognitive status, and clinical analysis of older persons in a long-term care facility in Chile, before and following the outbreak of COVID-19. **Methods.** A single-center, pre–post, and Pearson’s correlational study was conducted in a cohort of 20 persons positive for COVID-19 from a total of 45 residents. Data on demographic, clinical, functional (Barthel Index (BI) and Katz) and cognitive (Mini mental Examination) status, and physiological function (hematology, lipidic and biochemical profiles) were collected. **Results.** The mean age was 84 ± 2.4 years, and 80% were females. The most common comorbidities were Arterial Hypertension, Diabetes Mellitus type II, and Alzheimer’s disease. Physical frailty was confirmed by body weight, body mass index, and calf circumference. Pre-infection, BI was negatively correlated with lipidic profile and erythrocyte sedimentation rate (ESR), and positively with frailty (calf circumference). Pre–post analysis showed that frailty and most analytical results were not modified. However, functional dependence on daily live activities significantly increased as measured by BI, with worse grooming and bowel and bladder controls. Post-infection, correlations were lost except between BI and ESR, and decreased albumin levels were found. **Conclusions.** The worsening of specific functional limitations emphasizes the need for targeted interventions that can be correlated with ESR. Albumin appears as a potential biomarker for physiological dysfunction associated with their infectious/inflammatory processes.

## 1. Introduction

Frailty corresponds to a syndrome of well-defined biological and clinical characteristics within its physical phenotype; it is multidimensional, dynamic, and nonlinear [1]. It increases after 65 years of age, being highly prevalent in older people [1,2]. Frailty syndrome can be considered as a state of pre-disability or risk of developing disability and dependence from a situation of incipient functional limitation. It is identified by a decrease in resistance and physiological reserves that lead to wear and tear of physiological systems, causing adverse health effects [1,3].

Frailty is a clinical condition that focuses on the high risk of developing negative health-related events due to the individual’s excessive vulnerability to both endogenous and exogenous stressors [1,2]. Frailty is a common and clinically significant condition among older people. Adverse health outcomes, including hospitalization, falls, disability, and mortality, significantly increase when comorbidities, low physical activity, poor dietary intake, and low socioeconomic status are present, among several other factors [4].

In this context, older people living in long-stay facilities have the highest indicators of frailty and disability to some degree, with the presence of chronic noncommunicable diseases [5]. This group of people, in addition, is susceptible to contracting COVID-19 and being affected to a greater extent by the consequences of the pandemic in multiple dimensions. Understanding how well older people function in basic daily activities and their level of disability and dependency and an assessment of biomarkers could highlight the actual health issues they face and their susceptibility to emerging diseases like COVID-19.

Recent reports have stated that frailty has been identified as a potential determinant of increased vulnerability in older people affected by COVID-19, as it may suggest alterations in physical performance and functional autonomy [6,7,8].

On the other hand, post-COVID syndrome after mild infection may result in impaired bodily functions and activities. The COVID-19 pandemic still generates many affected patients and a significant mortality rate [9]. In addition, evidence indicates that various age- and frailty-related diseases are modulated by physical activity and social engagement, both of which are limited in confinement situations such as those occurring in periods of confinement and quarantine.

The present work aims to determine the frailty status and functional limitations and clinical analysis of older persons following an outbreak of COVID-19 in a long-stay facility to identify the specific consequences and needs of this population to ensure their inclusion in public health recommendations and consideration by policymakers.

## 2. Materials and Methods

### 2.1. Study Design and Participants

A single-center pre–post analysis and correlational study was conducted among residents of the long-stay facility in Chile. A review of 45 clinical records and health reports from the Ministry of Health Secretariat (SEREMI) of the La Araucanía region was conducted October 2022 and March 2023. A positive COVID-19 test was considered an inclusion criterion, along with being over 65 years of age and a permanent resident of the long-stay facility. The lack of clinical records of the study variables was considered an exclusion criterion.

A total of 20 residents’ positives to COVID-19 were included, with 16 (80%) women and 4 (20%) men. The mean age was 84 years (minimum 60, maximum 97) with a mean of 5 years of residence in the facility. All positive cases of COVID-19 were diagnosed according to established criteria (WHO interim guidance) by a reverse transcriptase Real Time polymerase chain reaction (RT-PCR) assay of nasal and pharyngeal swab specimens. All residents completed a quarantine period of one month and one week and remained in the facility with routine monitoring for signs and symptoms, support with activities of daily living, and regularly assisted ambulation when possible. A PCR test was applied to assess the end of quarantine and detect new cases. The infection period for 18 residents was in July–August, and two points between February and April 2022. Two deaths were recorded during 2022, but neither case was directly related to the period of SARS-CoV-2 infection.

### 2.2. Data Collection

A review was carried out of the entire population belonging to the long-stay facility. A total of 45 medical records were included between October 2022 and March 2023, with 20 cases of SARS-CoV-2 being recorded in 2022.

For the positive cases included in the study, epidemiological, demographic, and clinical data were obtained from the history of each patient. In addition, operational test data were recorded: disability level and functional status (Barthel Index and Katz index), cognitive status (Mini mental), frailty phenotype and physiological function (hematology, lipid, and chemistry lab work). The laboratory tests were obtained, including biochemical profile and blood count data. The hematologic results were obtained by complete blood count through hematology analyzer MINDRAY CAL 6000 (Mindray, Shenzhen, China), obtaining the neutrophil–lymphocyte ratio (NLR) and platelet–lymphocyte ratio (PLR). The functional tests included in the study were performed by the health care team of the facility, and six months before infection and six months after infection were defined to identify functional, frailty, and laboratory test results. In addition, vaccines and doses administered before the positive cases were recorded.

### 2.3. Statistical Analysis

The quantitative variables results are presented as mean, standard deviation, and SEM. In the case of nominal and categorical qualitative variables, they are presented as percentages and proportions. The relationship and associativity between the functionality, frailty, and physiological function variables were examined through the one-sample paired *t*-test. The variables that did not present a normal distribution were normalized with a square root or were fractionated with Fractional rank [10].

Additionally, a correlation of the functionality and frailty parameters with the most significant biological parameters obtained in the laboratory tests was performed using Pearson’s correlation, and Point-Biserial Correlation. For all analyses, a two-tailed *p*-value < 0.05 was considered statistically significant. All analyses were performed with SPSS 15.0 software (SPSS Inc., Chicago, IL, USA).

### 2.4. Ethics

The ethical guidelines for research defined by the Declaration of Helsinki, supported by the good clinical practice guidelines for managing anonymized data from the source, were considered. The informed consent was approved by the Ethics Committee of the Universidad Católica de Temuco, CEIUCT0411003/23. Also, the residents’ relatives gave their approval for the development of the study through a written manifestation.

## 3. Results

### 3.1. Baseline Characteristics

The baseline characteristics of the cohort before SARS-CoV-2 infection are shown in Table 1. A total of 20 residents were included, of whom 16 (80%) were women and 4 (20%) were men. The mean age was 84 ± 2.42 years (minimum 60, maximum 97) with a mean of 5 ± 0.72 years of residence in the facility. A total of 80 percent of the sample had Arterial Hypertension (16/20), followed by Diabetes Mellitus type II with 40% (8/20) and Alzheimer’s disease with 30% (6/20). The level of disability recorded by the Chilean National Disability Service (SENADIS) indicated that 72% of the cohort had some degree of disability, 30% being physical (6/20), 25% mental (5/20), and 35% (7/20) of mixed type (physical and mental). In addition, the Pfeiffer questionnaire, used for the detection and degree of cognitive impairment, indicated severe impairment in 45% (9/20) of the cohort, with only 15% (3/20) of normal cognitive status.

On the other hand, the residents had their complete vaccination schedule, with 100% in the first and second doses (CoronaVac schedule, according to the regulations in force in the country by the Ministry of Health, MINSAL) and 95% and 90% in the third and fourth doses.

### 3.2. Frailty and Functional Limitation

Table 2 shows the results obtained in the physical frailty and functionality conditions pre- and post-infection. No statistically significant differences were observed in the physical frailty variables, but significant differences were observed in the functionality variables measured in Barthel Index (BI). Thus, differences in the total score indicated an increase in functional dependence on basic activities of daily living (ADL) [*t* = 2.220, *p* = 0.039; CI: 0.612–20.887]. In addition, the indicators of grooming, bowel control, and bladder control of the BI showed significant changes post-infection, with a higher degree of functional dependence associated with disability [Grooming, *t* = 2.854, *p* = 0.010; CI: 0.399–2.600. Bowel control, *t* = 2.101, *p* = 0.049; CI: 0.006–3.493. Bladder control, *t* = 2.373, *p* = 0.028; CI: 0.235–3.764].

### 3.3. Physiological Fuction

On the other hand, when comparing the results of physiological function pre- and post-SARS-CoV-2 infection, we observed significative differences for albumin [4.213 + 0.1727 g/dL pre-infection vs. 3.417 + 0.7441 post-infection; *p* < 0.05], see Table 3. Also, in pre-SARS-CoV-2 infection, a negative correlation was found between Barthel Scale/Index (BI) and physiological function as measured by cholesterol [r^2^ = (−) 0.421, *p* = 0.006], HDL cholesterol [r^2^ = (−) 0.339, *p* = 0.018], LDL cholesterol [r^2^ = (−) 0.323, *p* = 0.021], triglycerides [r^2^ = (−) 0.355, *p* = 0.019], and erythrocyte sedimentation rate [r^2^ = (−) 0.465, *p* = 0.007]. A positive correlation was also found between BI and frail status measured by calf circumference [r^2^ = 0.270, *p* = 0.039]. In post-SARS-CoV-2 infection patients, a negative correlation was found between BI and the erythrocyte sedimentation rate [r^2^ = (−) 0.367, *p* = 0.022].

## 4. Discussion

### 4.1. Frailty and Functional Limitation

The MMSE test did not reflect the severe cognitive status (Pfeiffer, dementia) of 9 of the 20 individuals. Regrettably, the MMSE test excluded 4 individuals due to their severe dementia stage, as the test’s length and complexity necessitated the use of pencil and paper, unlike the Pfeiffer test. In addition, the Barthel Index provides a more detailed assessment of motor skills, while the Katz Index focuses more on the ability to perform complex tasks. The latter already showed a significant deterioration before COVID-19 infection, so the Katz test reported no changes, unlike the Barthel Index, which indicated a greater physical decline.

Frailty may have contributed to the vulnerability of older people to a more severe clinical presentation during the COVID-19 pandemic [11]. Studies have shown that older COVID-19 patients with a frailty condition have an increased risk of mortality compared with non-frail patients, and this association is independent of other clinical and demographic factors [7,11]. Frailty should be part of the risk assessment of older people with COVID-19 [12].

The clinical frailty scale (CFS) is a tool used to assess frailty in COVID-19 patients [13]. Studies have shown that a severe degree of frailty among COVID-19 patients was associated with prolonged hospitalization, all-cause mortality, and higher mortality risk in the next 2 weeks following discharge [8]. In addition, a study in Italy found that one in three older patients previously hospitalized for COVID-19 had an unfavorable transition in CFS score during a median follow-up of almost 6 months [14]. Although frailty has been endorsed as a tool to inform estimates of COVID-19 risk, studies suggest it may have a broader role in primary care and public health by identifying people who may benefit from interventions to reduce health and social impacts of COVID-19 and future pandemics [15].

The results show that the majority of patients who had COVID-19 did not present severe or severe symptoms regardless of age, comorbidities, or frailty. A retrospective population-based cohort study in Canada showed that persons not vaccinated with COVID-19 had a 10-fold and 21-fold increased risk of hospitalization relative to whether they had been fully vaccinated or boosted, resulting in many hospital bed days and avoidable costs. [16]. Limited evidence suggests that the pre-vaccination COVID-19 status was the only factor associated with vaccine immunogenicity, but not age, frailty, or disability [17]. However, a recent review on the interrelationships between frailty and immunity in older people showed that a well-functioning immune system prevented frailty and vice versa, and adherence to an immunization schedule prevented frailty and maintained immune homeostasis [17]. Frail older people and those in deprived areas remain at risk of severe COVID-19 outcomes, and the vaccine has been shown to reduce the severity of COVID-19 and the chances of long-term symptoms [18]. Recent evidence indicates that frailty can independently predict the reduced antibody response after COVID-19 mRNA vaccines in older people [19]. While the coming COVID-19 vaccination on frail older people is challenging because they may be benefited and harmed by the newly developed vaccines, there is hope that the vaccine can help reduce the severity of COVID-19 outcomes in frail older people [20].

### 4.2. Physiological Function

Clinical analysis of aging and frailty may predict COVID-19 severity and poor prognosis in older people [21]. Various authors have proposed a spectrum of candidate biomarkers for the illnesses associated with COVID-19, aging, and frailty [21]. Thus, the alteration of inflammatory biomarkers, lactate dehydrogenase, procalcitonin, and vitamin D levels found in the present study might identify individuals with poor COVID-19 prognoses [21]. A retrospective cohort study in patients aged 60 years or older who were hospitalized with COVID-19 at an intensive care unit showed that inflammatory biomarkers like the neutrophil–lymphocyte ratio, procalcitonin, C-reactive protein (CRP), and d-dimer were associated with mortality in older patients with COVID-19 [22]. Also, a recent cross-sectional analysis of 202,537 participants in the UK Biobank demonstrated an association of several biomarkers with pre-frailty and frailty, reporting that patients with frailty show changes in physiological function, with lower levels of apoA1, LDL, and HDL cholesterol, albumin, vitamin D, total bilirubin, apoB, and testosterone. On the other hand, higher concentrations of triglycerides, Gamma-glutamyl Transferase (GGT), cystatin C, CRP, Alkaline phosphatase (ALP), and phosphate were observed [23]. When comparing the results of physiological function pre- and post-SARS-CoV-2 infection, we observed significative differences for albumin. This finding could be related to pathophysiological processes in liver function, which correlates with previous reports of lower serum albumin concentration in patients with frailty [23]. In addition, although no significant changes were observed in ALP concentrations pre- and post-infection by SARS-CoV-2, there is a tendency to present ALP elevation, especially post-infection (162.6 ± 39.31 U/L).

Additionally, the potential association between functional status was investigated in the frailty group. The results show a negative correlation between physiological function and Barthel Index in pre-SARS-CoV-2 infection in frailty patients for total cholesterol, HDL cholesterol, LDL cholesterol, triglycerides, and erythrocyte sedimentation rate. In post-SARS-CoV-2 infection patients, a negative correlation was found between the erythrocyte sedimentation rate and Barthel Index. Thus, the higher the Barthel Index, the greater the dependence on activities of daily living. Regarding HDL cholesterol, the results show HDL cholesterol levels below the reference range, which would correlate with higher requirements for follow-up and monitoring of activities of daily living in the subjects included in the study. Previous reports show that high HDL cholesterol levels constitute a protective factor against daily living disability and correlate with better functional status [24,25]. A recent report, including 822 females in China, showed that the association of higher normal lipid profile indicators, especially HDL cholesterol, might have a protective effect on ADL disability among female centenarians [24]. Studies have described the relationship between inflammatory biomarkers and frailty [26,27]. There were no significant differences in ESR and NLR in the pre- and post-infection measurements in the patient group. However, a negative correlation was observed between ESR and ADL scores in pre- and post-infection measurements. Previously, a cross-sectional study in Tunisian older people showed a significant negative correlation between inflammatory physiological function with disability [28]. Regarding the finding of a negative correlation between ESR and the Barthel Index, future work is required to understand this association.

Our study has some limitations. First, a limited number of residents were included, so the results should be contrasted with a larger and more diverse cohort of patients. Second, it was impossible to determine those negative in the PCR test because only the clinical records of the positive cases were authorized for review; it would be relevant in future research to have this information. Finally, studies suggest that malnutrition is closely related to frailty, showing that frail patients have significantly lower levels of prealbumin compared to non-frail subjects [29]. In our study, this biomarker was not measured, so its measurement would be valuable in subsequent studies.

## 5. Conclusions

In conclusion, the results of this study allow us to characterize several relevant aspects: a state of pre-existing frailty, comorbid with poor cognitive status of older people (Alzheimer’s disease), Arterial Hypertension and Diabetes Mellitus type II, and functional disability. Correlation analysis between frailty, functional and physiological status indicated a pre-existing positive correlation between Barthel Index (BI) and frail status (calf circumference) but negative correlation with physiological functions (HDL and LDL cholesterol, and triglycerides). Before and after the infection, BI was negatively correlated with the erythrocyte sedimentation rate. Infection by SAR-CoV-2 worsened the ADL functional capacities as measured by BI and also reduced albumin levels, which could be a potential biomarker related to hepatic physiological changes associated with their infection/inflammatory processes.

## Figures and Tables

**Table 1:** Baseline characteristics of demographic and clinical data of the cohort before SARS-CoV-2 infection.

Parameters	Group	Females	Males
Sex	20 (100%)	16 (80%)	4 (20%)
Morbidity			
Arterial Hypertension	16 (80%)	12 (75%)	4 (25%)
Diabetes Mellitus type II	8 (40%)	6 (75%)	2 (25%)
Alzheimer’s disease	6 (30%)	6 (100%)	-
Stroke Sequel	5 (25%)	2 (40%)	3 (60%)
Heart disease	3 (15%)	3 (100%)	-
Chronic Obstructive Pulmonary Disease	3 (15%)	2 (67%)	1 (33%)
Level of disability (%)	18 (72%)	14 (80%)	4 (20%)
Physical	6 (30%)	3 (21%)	3 (75%)
Cognitive	5 (25%)	5 (36%)	-
Physical and cognitive	7 (35%)	6 (43%)	1 (25%)
Cognitive impairment (Peiffer)			
Normal	3 (15%)	1 (15%)	2 (50%)
Mild	5 (25%)	5 (25%)	-
Moderate	3 (15%)	2 (15%)	1 (25%)
Severe	9 (45%)	8 (45%)	1 (25%)
Vaccination			
1st dose	20 (100%)	16 (100%)	4 (100%)
CoronaVac (Sinovac)	20 (100%)	16 (100%)	4 (100%)
2nd dose	20 (100%)	16 (100%)	4 (100%)
CoronaVac (Sinovac)	20 (100%)	16 (100%)	4 (100%)
3rd dose	19 (95%)	16 (100%)	3 (75%)
ChAdOx1 nCoV-19 (AstraZeneca)	11 (58%)	9 (56%)	2 (67%)
BNT162b2 (Pfizer-BioNtech)	7 (37%)	6 (38%)	1 (33%)
CoronaVac (Sinovac)	1 (5%)	1 (7%)	-
4th dose	18 (90%)	15 (94%)	3 (75%)
BNT162b2 (Pfizer-BioNtech)	18 (100%)	15 (100%)	3 (100%)

**Table 2:** Physical frailty conditions and functionality.

	Pre-Infection	Post-Infection
	Mean ± SEM	Mean ± SEM
Physical frailty		
Age (years)	84 ± 2.42	**-**
Years of residence	5 ± 0.72	**-**
Body weight	56.11 ± 2.87	55.83 ± 2.96
BMI	23.13 ± 1.03	23 ± 4.64
Calf circumference	29.94 ± 1.12	29.42 ± 1.02
Functionality (ADL)		
Barthel Scale/Index (BI)		
Total	44 ± 7.04	33 ± 7.85 *
Feeding	7 ± 0.84	6 ± 1.02
Bathing	2 ± 0.53	1 ± 0.51
Dressing	3 ± 0.91	3 ± 0.92
Grooming	2 ± 0.57	1 ± 0.41 *
Bowel control	7 ± 0.98	5 ± 1.03 *
Bladder control	6 ± 1.02	4 ± 1.04 *
Toilet use	3 ± 0.99	3 ± 0.99
Transfers	6 ± 1.10	5 ± 1.17
Mobility	6 ± 1.37	5 ± 1.30
Stairs	2 ± 0.57	2 ± 0.53
Katz	4 ±0.47	4 ± 0.51
Cognitive impairment		
Mini mental	14 ± 1.78	15 ± 0.79

(*) represent the significant differences (*p* < 0.05) using Student’s *t*-test.

**Table 3:** Clinical analysis (physiological function) pre- and post-SARS-CoV-2 infection in frailty patients.

Parameter	Reference	Pre-Infection	Post-Infection	Change
Hematology				
Hematocrit, %	35.0–47.0	38.17 ± 4.930	36.37 ± 4.841	+1.8
Hemoglobin, g/dL	13.0–17.5	12.92 ± 1.844	12.17 ± 1.763	−0.8
WBC, 10 × 10^3^ uL	4.00–12.00	8.578 ± 2.852	8.120 ± 2.492	−0.458
PLT, 10 × 10^3^ uL	150–450	215.89 ± 139.13	238.00 ± 74.40	+22.11
VHS, mm/h	<20	31.62 ± 22.13	35.36 ± 32.82	+35.36
NLR	0.107–3.19	3.594 ± 3.847	3.627 ± 3.129	+33.0
PLR	46.79–218.01	126.7 ± 132.1	148.9 ± 90.93	+22.2
Lipid profile				
Total cholesterol, mg/dL	<200	149.2 ± 34.58	145.3 ± 44.68	−3.9
HDL cholesterol, mg/dL	≥60	48.08 ± 12.60	45.82 ± 14.10	−2.26
LDL cholesterol, mg/dL	<100	79.11 ± 32.50	74.48 ± 35.55	−4.62
VLDL cholesterol, mg/dL	<30	27.97 ± 27.38	24.96 ± 16.79	−3.01
Triglycerides, mg/dL	<150	96.01 ± 43.33	106.9 ± 53.34	+10.89
Biochemistry				
Glucose, mg/dL	70–99	101.9 ± 22.10	109.3 ± 34.78	+7.4
Creatinine, mg/dL	0.60–1.20	0.83 ± 0.28	0.78 ± 0.25	−0.04
MDRD4, mL/min/1.73 m^2^	>60	83.80 ± 40.30	81.44 ± 27.41	−5.36
Urea, mg/dL	8–25	47.78 ± 20.96	41.14 ± 16.71	−6.64
Alkaline Phosphatase U/L	45–115	124.3 ± 48.70	162.6 ± 39.31	+38.3
Uric Acid, mg/dL	2.3–6.6	4.398 ± 2.545	3.920 ± 1.363	−0.478
Albumin, g/dL	3.5–5.0	4.213 ± 0.1727	3.417 ± 0.7441 *	−0.796
Sodium, mEq/L	135–145	140.4 ± 3.524	139.5 ± 2.841	−0.9
Potassium, mEq/L	3.5–5.0	4.441 ± 0.5161	4.313 ± 0.3681	−0.128
Chlorine, mEq/L	100–108	99.19 ± 4.334	101.2 ± 2.868	+2.01

Abbreviations: WBC = white blood cell count; PLT = platelets; ESR = erythrocyte sedimentation rate; NLR = neutrophil–lymphocyte ratio; PLR = platelet–lymphocyte ratio. (*) represent the significant differences (*p* < 0.05) using Student’s *t*-test.

## Data Availability

The original data presented in the study are openly available in the research repository of the Catholic University of Temuco.

## References

[1] Fried L.P., Tangen C.M., Walston J., Newman A.B., Hirsch C., Gottdiener J., Seeman T., Tracy R., Kop W.J., Burke G. (2001). Frailty in Older Adults: Evidence for a Phenotype. J. Gerontol. Med. Sci..

[2] Proietti M., Cesari M. (2020). Frailty: What Is It?. Adv. Exp. Med. Biol..

[3] Tabue-Teguo M., Simo N., Harmand M.G.C., Cesari M., Avila-Funes J.A., Féart C., Amiéva H., Dartigues J.F. (2017). Frailty in elderly: A brief review. Geriatr. Psychol. Neuropsychiatr. Vieil..

[4] Doody P., Lord J.M., Greig C.A., Whittaker A.C. (2023). Frailty: Pathophysiology, Theoretical and Operational Definition(s), Impact, Prevalence, Management and Prevention, in an Increasingly Economically Developed and Ageing World. Gerontology.

[5] Espinoza S.E., Quiben M., Hazuda H.P. (2018). Distinguishing Comorbidity, Disability, and Frailty. Curr. Geriatr. Rep..

[6] Holland C., Garner I., Simpson J., Eccles F., Pardo E.N., Marr C., Varey S. (2021). Impacts of COVID-19 lockdowns on frailty and wellbeing in older people and those living with long-term conditions. Adv. Clin. Exp. Med..

[7] Salini S., Russo A., De Matteis G., Piccioni A., Della Polla D., Carbone L., Barillaro C., Landi F., Franceschi F., Covino M. (2022). Frailty in Elderly Patients with Covid-19: A Narrative Review. Gerontol. Geriatr. Med..

[8] Hussien H., Nastasa A., Apetrii M., Nistor I., Petrovic M., Covic A. (2021). Different aspects of frailty and COVID-19: Points to consider in the current pandemic and future ones. BMC Geriatr..

[9] De Smet R., Mellaerts B., Vandewinckele H., Lybeert P., Frans E., Ombelet S., Lemahieu W., Symons R., Ho E., Frans J. (2020). Frailty and Mortality in Hospitalized Older Adults With COVID-19: Retrospective Observational Study. J. Am. Med. Dir. Assoc..

[10] Templeton G. (2011). A Two-Step Approach for Transforming Continuous Variables to Normal: Implications and Recommendations for IS Research. Commun. Assoc. Inf. Syst..

[11] Maltese G., Corsonello A., Di Rosa M., Soraci L., Vitale C., Corica F., Lattanzio F. (2020). Frailty and COVID-19: A Systematic Scoping Review. J. Clin. Med..

[12] Lee Y.K., Motwani Y., Brook J., Martin E., Seligman B., Schaenman J. (2022). Predictors of COVID-19 outcomes: Interplay of frailty, comorbidity, and age in COVID-19 prognosis. Medicine.

[13] Hewitt J., Carter B., Vilches-Moraga A., Quinn T.J., Braude P., Verduri A., Pearce L., Stechman M., Short R., Price A. (2020). The effect of frailty on survival in patients with COVID-19 (COPE): A multicentre, European, observational cohort study. Lancet. Public Health.

[14] Ferrara M.C., Zarcone C., Tassistro E., Rebora P., Rossi E., Luppi F., Foti G., Squillace N., Lettino M., Strepparava M.G. (2023). Frailty and long-COVID: Is COVID-19 responsible for a transition in frailty status among older adults who survived hospitalization for COVID-19?. Aging Clin. Exp. Res..

[15] Griffith L.E., McMillan J., Hogan D.B., Pourfarzaneh S., Anderson L.N., Kirkland S., Basta N.E., van den Heuvel E., Raina P. (2022). Frailty and the impacts of the COVID-19 pandemic on community-living middle-aged and older adults: An analysis of data from the Canadian Longitudinal Study on Aging (CLSA). Age Ageing.

[16] Bagshaw S.M., Abbott A., Beesoon S., Bowker S.L., Zuege D.J., Thanh N.X. (2023). A population-based assessment of avoidable hospitalizations and resource use of non-vaccinated patients with COVID-19. Can. J. Public Health.

[17] Chen L.K. (2021). COVID-19 vaccination and frailty in older adults. Arch. Gerontol. Geriatr..

[18] Saul H., Gursul D., Antonelli M., Steves C. (2022). Frail older people and those in deprived areas remain at risk from covid-19, even after vaccination. BMJ.

[19] Kountouras J., Tzitiridou-Chatzopoulou M., Papaefthymiou A., Chatzopoulos D., Doulberis M. (2023). COVID-19 mRNA Vaccine Effectiveness against Elderly Frail People. Medicina.

[20] Rolland Y., Cesari M., Morley J.E., Merchant R., Vellas B. (2021). COVID19 Vaccination in Frail People. Lots of Hope and Some Questions. J. Nutr. Health Aging.

[21] Wanhella K.J., Fernandez-Patron C. (2022). Biomarkers of ageing and frailty may predict COVID-19 severity. Ageing Res. Rev..

[22] Vieira J.A.R., da Costa R.S., Monteiro J.M., Alves J.A.R., Spinassé C.M., Pupim C.T., Tieppo A., Morelato R.L. (2022). Association of inflammatory and coagulation biomarkers with mortality in patients aged 60 years or older and hospitalized with COVID-19. Geriatr. Gerontol. Aging.

[23] Chu W., Lynskey N., Iain-Ross J., Pell J.P., Sattar N., Ho F.K., Welsh P., Celis-Morales C., Petermann-Rocha F. (2023). Identifying the Biomarker Profile of Pre-Frail and Frail People: A Cross-Sectional Analysis from UK Biobank. Int. J. Environ. Res. Public Health.

[24] Wang S., Liu M., Yang S., Wang J., Jia W., Cao W., Han K., He Y. (2020). Higher Normal Levels of Triglyceride and Low and High-Density Lipoprotein Cholesterol Might Have a Protective Effect Against Activities of Daily Living Disability Within Chinese Female Centenarians: A Cross-Sectional, Complete Sample Study. Clin. Interv. Aging.

[25] Formiga F., Ferrer A., Chivite D., Pinto X., Cuerpo S., Pujol R. (2011). Serum high-density lipoprotein cholesterol levels, their relationship with baseline functional and cognitive status, and their utility in predicting mortality in nonagenarians. Geriatr. Gerontol. Int..

[26] You L., Bai C., Wang S., Yu X., Wang T., Lu M., Tan C., Guan Y. (2023). The Prevalence and Factors That Impact Frailty in Inflammatory Bowel Disease Patients Hospitalized in a Tertiary Hospital in China. Dig. Dis..

[27] Soysal P., Stubbs B., Lucato P., Luchini C., Solmi M., Peluso R., Sergi G., Isik A.T., Manzato E., Maggi S. (2016). Inflammation and frailty in the elderly: A systematic review and meta-analysis. Ageing Res. Rev..

[28] Hammami S., Ghzaiel I., Hammouda S., Sakly N., Hammami M., Zarrouk A. (2020). Evaluation of pro-inflammatory cytokines in frail Tunisian older adults. PLoS ONE.

[29] Xue Q.L. (2011). The Frailty Syndrome: Definition and Natural History. Clin. Geriatr. Med..

